# Effectiveness of Ayurvedic Nutritional Supplements and Yoga Protocol in Reducing the Incidence and Severity of Acute Mountain Sickness (AYAMS Study): Study Protocol for an Open-Label Randomized Controlled Trial

**DOI:** 10.2196/81567

**Published:** 2026-04-08

**Authors:** Amit Kumar Rai, Sophia Jameela, Arun Kumar Yadav, Rajneesh Kumar Joshi, Pallavi Mundada, Azeem Ahmad, Bidhan Mahajon, Bhagwan Sahai Sharma, Shruti Khanduri, Babita Yadav, Arunabh Tripathi, Rakesh Kumar Rana, Bhogavalli Chandrasekhararao, Narayanam Srikanth, Raghavendra Rao, Rabinarayan Acharya

**Affiliations:** 1 Ayurvedic and Unani Tibbia College and Hospital, New Delhi, India; 2 Central Council for Research in Ayurvedic Sciences, New Delhi, India; 3 Office of the DGAFMS, Fifth Floor, Block A, Defence Officer Complex, Africa Avenue, New Delhi, 110023, India, 91 9868815430; 4 All India Institute of Ayurveda, New Delhi, India; 5 Central Council for Research in Yoga and Naturopathy, New Delhi, India

**Keywords:** acute mountain sickness, acclimatization, high altitude, *Terminalia arjuna*, *Withania somnifera*, yoga

## Abstract

**Background:**

Acute mountain sickness (AMS) poses a unique and formidable challenge to healthy personnel at high altitudes.

**Objective:**

This randomized controlled trial (RCT) protocol aimed to assess the effectiveness of Ayurvedic nutritional supplements in conjunction with a yoga protocol in reducing the incidence and severity of AMS among healthy personnel stationed in the challenging high-altitude landscapes of the western Himalayas.

**Methods:**

The proposed open-label, parallel-group RCT was conducted in apparently healthy individuals of any gender aged 18‐50 years. The study was conducted at two distinct high-altitude stages within the western Himalayan region: stage I, situated at an elevation ranging from 9000 to 12,000 feet and stage II, spanning 12,000 to 15,000 feet. A total of 1660 participants (n=830 per stage) underwent random assignment in a 1:1 ratio to receive either the existing acclimatization schedule (AS) for high altitude (control group) or the Ayush intervention (Ayush group) in addition to the AS. The participants in the Ayush group received Ayurvedic nutritional supplements, including the Ayur-nutri kit (Ayush poshak yoga, 25 g, and Ayush cardiac care tea, 125 mL), twice daily, along with a yoga protocol (60 min daily) for 120 days. The primary outcome was the incidence of AMS, assessed using the 2018 Lake Louise Scoring System, and the proportion of participants with a Lake Louise Scoring System score of 6 or higher during the first 7 days from baseline. The secondary outcome measures included the proportion of participants with thromboembolic events; changes in coagulation and hemostasis activation markers and proinflammatory markers; and changes in self-reported negative emotional states (depression, anxiety, and stress), sleep quality, and overall quality of life (assessed through Depression Anxiety Stress Scale-21 items, Pittsburgh Sleep Quality Index, and 12-item short-form, respectively) on day 60 and day 120 from baseline.

**Results:**

The study was funded in March 2023. The data collection was completed in December 2023. A total of 1660 participants were enrolled in the study. The analysis of the study data is in progress. The study outcomes are expected to be published by December 2026.

**Conclusions:**

This RCT was the first of its kind to explore the potential benefits of using Ayurvedic nutritional supplements and a yoga protocol in conjunction with the standard AS to reduce the occurrence and severity of AMS among healthy personnel. The outcomes of this trial can aid in better acclimatization and resilience among healthy personnel at high altitudes.

## Introduction

### Background

High-altitude illness (HAI) is the collective term used for various debilitating health conditions that can affect individuals who are not acclimatized and ascend rapidly to high altitudes within 6 to 12 hours of their ascent to high altitude [1,2]. Conventionally, 2500 meters has been used as the threshold for HAI [2]. The spectrum of HAI encompasses various conditions that can range from mild to severe, among which the most typical are acute mountain sickness (AMS), high-altitude cerebral edema, and high-altitude pulmonary edema (HAPE). High-altitude cerebral edema and HAPE occur much less frequently than AMS but are potentially fatal. The risk factors for HAI are the rate of ascent, high altitude reached (especially the sleeping altitude), previous high-altitude exposure, and individual susceptibility [1,3,4]. AMS is a common clinical presentation in individuals venturing over 2500 meters and above [5]. The prevalence and severity of AMS rise with increasing altitude. It occurs in approximately 10% to 25% of nonacclimatized individuals at 2500 meters, which may increase to 50% to 85% at an altitude of 4500 to 5500 meters [2,6-8]. The pathophysiology of AMS includes relative hypoventilation, impaired gas exchange, increased sympathetic activity, fluid retention and redistribution, and raised intracranial pressure in case of moderate to severe AMS [1,5]. AMS presents with a group of nonspecific symptoms such as headache (primary defining symptom), gastrointestinal symptoms (anorexia, nausea, or vomiting), fatigue and/or weakness, dizziness, or disturbed sleep [9].

A gradual ascent to enable acclimatization is usually impractical logistically for individuals susceptible to AMS, particularly in recreational, tactical, and exigency situations. Considering this limitation, several clinical studies have been conducted over the last 2 decades to explore the efficacy of conventional medications in reducing the incidence of AMS, followed by a few systematic reviews [10-13]. The interventions, such as acetazolamide, dexamethasone, and ibuprofen, have shown promising results in reducing the severity of AMS; however, they have specific adverse effects, limiting their routine use. Acetazolamide has reported adverse effects, including headache, nausea, polyuria, and dysgeusia [11,12]. Similarly, dexamethasone and ibuprofen have shown adverse effects such as gastrointestinal symptoms, headache, skin rashes, edema, and mood changes (including depression) [12]. Considering this, exploring effective, safe, and readily available interventions for prophylaxis and management of AMS is essential.

Ayurveda, the traditional Indian medicine, aims to improve the quality and span of life, primarily emphasizing disease prevention and health promotion by strengthening body tissues to withstand both exogenous and endogenous stressors. The same can be achieved by modulating diet and lifestyle and by using Ayurvedic nutritional supplements (containing herbal ingredients) such as *Rasayana*, which help restore the body’s equilibrium. The herbal ingredients used in Ayurvedic nutritional supplements are known for their adaptogenic, antioxidant, and immunomodulatory properties [14-16]. A holistic approach involving the use of Ayurvedic nutritional supplements and a yoga protocol may have significant scope for reducing AMS, effectively addressing psychological and physiological well-being, considering that while the Ayurveda supplements enable the body to cope with the physical stressors, the yoga protocol can enhance the mental resilience and focus, which are crucial for operating in challenging conditions.

### Rationale for Study Interventions

The Ayur-nutri kit comprises Ayush poshak yoga, a nutritional supplement, and Ayush cardiac care tea (CCT), an herbal drink, having ingredients such as *Terminalia arjuna* (Roxb.) Wight & Arn*, Crocus sativus* L.*, Withania somnifera* (L.) Dunal*, Tinospora cordifolia* (Willd.) Miers ex Hook.f*, Zingiber officinale* Roscoe*, Piper longum* L.*, Piper nigrum* L.*, Chlorophytum borivilinium* Santapau & R.R.Fern., etc, along with a wide variety of nuts and seeds such as almonds, walnuts, cashews, pistachios, pumpkin seeds, and melon seeds. The herbs in these nutritional supplements have cardioprotective, neuroprotective, hepatoprotective, antihypertensive, antidyslipidemic, anti-inflammatory, antiatherogenic, antiplatelet aggregation, antithrombotic, and anticoagulant activities [17-21]. A clinical study on these nutritional supplements, conducted among volunteers of the Antarctica expedition, reported significant improvements in stress-related changes in immunoglobulins and antioxidant enzymes, alongside an enhanced quality of life. This suggests that they are effective in managing cold stress and were also found to be safe [22]. Preliminary studies have shown that yoga contributes to acclimatization at high altitude and plays a crucial role in maintaining physical fitness and mental resilience [23]. Respiratory adaptations induced by yoga may offer an efficient strategy to cope with high altitude–induced hypoxia [24,25].

Based on the evidence stated above, this study has been conceptualized to assess the comparative effectiveness of incorporating a combined protocol of Ayurvedic nutritional supplements and yoga alongside the existing acclimatization regimen for preventing AMS among healthy personnel at high-altitude terrains.

### Objectives

The present randomized controlled trial (RCT) was designed to assess the effectiveness of a combination of Ayurvedic nutritional supplements and yoga protocol in reducing the incidence and severity of AMS compared to the standard acclimatization schedule (AS) at high-altitude areas in the western Himalayan region. The key secondary objectives of this study included evaluating the impact of the Ayush intervention protocol (Ayur-nutri kit + yoga) on subjective and assessment parameters, including coagulation and hemostasis activation markers, proinflammatory markers, negative emotional states (depression, anxiety, and stress), sleep quality, and overall quality of life after 120 days. Another secondary objective was assessing the safety of Ayush interventions in AMS.

## Methods

### Study Design and Setting

The study protocol has been drafted in accordance with the Standard Protocol Items: Recommendations for Interventional Trials (SPIRIT) guidelines [26]. This clinical study was an open-label, randomized, controlled parallel-group trial. High-altitude areas are classified into three stages: stage I (9000‐12,000 feet), stage II (12,000‐15,000 feet), and stage III (more than 15,000 feet) [27]. The study was conducted at stage I and II high-altitude areas in the western Himalayan region.

### Study Participants

Apparently healthy personnel of any gender aged 18‐50 years, deemed physically fit for sojourn to high-altitude areas (in the western Himalayan regions) and willing to provide written informed consent, were included in the study. The study population consisted of individuals who were sojourning at high altitude for the first time and those who resumed sojourns after a break.

Individuals with a history of underlying cardiovascular disease or a clinically significant electrocardiogram abnormality, cerebrovascular disease, pulmonary disease, neurological disease, psychiatric illness, major venous thromboembolism (deep vein thrombosis, pulmonary embolism, or cerebrovascular thrombosis), sickle-cell disease, metabolic disorders, malignancy, glaucoma; a history of pulmonary edema or severe physiological response to high altitude (such as HAPE); evidence of uncontrolled diabetes mellitus (glycated hemoglobin more than 8.0%), uncontrolled hypertension (more than 140/90 mm Hg) even after medications, abnormal hepatic function (aspartate transaminase and/or alanine transaminase more than 2 times the upper limit of normal), or abnormal renal function (serum creatinine more than 1.2 mg%); or BMI ≥30 kg/m^2^ were excluded from the study. Similarly, those with a history of any surgery within the past 6 months; transfusion of blood or any blood product within 90 days before screening; regular use of any concomitant conventional medication; a history of chain smoking, alcohol use disorder, or substance abuse; and the presence of any other clinical condition which the investigator thinks may jeopardize the safety of the study participants were not considered for the study.

### Study Intervention

The eligible participants in the Ayush group (AG) received a comprehensive Ayush-AMS protocol, which included the use of an Ayur-nutri kit and a yoga protocol, along with the existing AS, for 120 days while staying in high-altitude regions [27].

The Ayur-nutri kit contains 2 herbal products, viz, an Ayurvedic nutritional supplement, Ayush poshak yoga, and an herbal tea, Ayush CCT, developed by the Central Council for Research in Ayurvedic Sciences, Ministry of Ayush, Government of India, to address nutritional supplementation requirements and to maintain healthy well-being at high-altitude regions. The Ayush poshak yoga powder contains a diverse blend of nuts and seeds, including cashews, almonds, pistachios, walnuts, pumpkin seeds, and cucumber seeds. It also contains herbs such as *W. somnifera* (L.) Dunal, *P. longum* L., *T. cordifolia* (Willd.) Miers ex Hook.f, *P. nigrum* L.*, Z. officinale* Roscoe., *Foeniculum vulgare* Mill., renowned for their nutritional and therapeutic benefits (Table 1). The Ayush CCT is a novel herbal tea that combines the heritage of traditional tea with a blend of herbal ingredients, including *T. arjuna* (Roxb.). Wight & Arn, saffron, cardamom, and cinnamon that can promote cardiovascular health, overall well-being, and healthy circulation (Table 1). Ayush CCT was made into an infusion by dipping one Ayush CCT tea bag in 125 mL of hot water for 5 minutes, allowing for an appropriate steeping time, and then used warm. Ayush poshak yoga was given in a dose of 25 g twice daily (morning and evening, 1 h before meals), along with 125 mL of Ayush CCT.

**Table 1.** Details of ingredients of Ayush poshak yoga and Ayush CCT^a^.

Ayur-nutri kit and ingredients (botanical/English name)	Part used
Ayush poshak yoga	
*Anacardium occidentale* L. (Cashew nut)	Kernel
*Prunus amygdalus* Stokes (Almond)	Kernel
*Pistacia vera* L. (Pistachio)	Kernel
*Juglans regia* L. (Walnut)	Cotyledons
*Papaver somniferum* L.	Seed
*Chlorophytum borivilianum* Santap & R.R.Frn	Tuberous root
*Cucumis melo* L.	Seed
*Cucumis sativus* L.	Seed
*Citrullus vulgaris* Schrad.	Seed
*Cucumis melo* var. *utilissimus* Dutt & Fueller	Seed
*Zingiber officinale* Rosc	Rhizome
*Piper longum* L.	Fruit
*Piper nigrum* L.	Fruit
*Withania somnifera* (L.) Dunal.	Root
*Foeniculum vulgare* Mill.	Fruit
*Tinospora cordifolia* (Willd.) Miers	Stem
Sugar candy	
Excipients, preservatives, flavoring agents (approved)	
Ayush CCT	
*Camellia sinensis* (L.) O.Kuntze	Leaf
*Elettaria cardamomum* Maton	Seed
*Cinnamomum verum* Breyn	Stem bark
*Crocus sativus* L. (Saffron)	Style and stigma
*Terminalia arjuna* (Roxb.) Wight & Am	Stem bark

a CCT: cardiac care tea.

The yoga protocol (High-Altitude Illness Prevention Protocol of Yoga) has been tailor-made for high altitude by the Central Council for Yoga & Naturopathy, Ministry of Ayush, Government of India. The participants performed the yoga protocol under the supervision of a qualified yoga instructor. It involved sessions beginning with breathing exercises, moving onward to performing Asanas in standing posture (05 Asanas), followed by Asanas in supine (05), prone (02), and sitting postures (02). The yoga session concluded with a sequence of Pranayama and meditation or breathing exercises, totaling approximately 60 minutes each day. The yoga schedule followed an alternating pattern throughout the 120 days (Table 2). Incorporating diverse asanas within varying postures, strategically arranged in specific patterns, ensured that each sequence effectively addressed certain aspects, such as the targeted engagement of distinct muscle groups and nurturing mental well-being.

**Table 2.** Details of yoga protocol (High-Altitude Illness Prevention Protocol of Yoga).

Type of yoga	Yoga practices (alternate day schedule)		Rounds	Duration^a^ (min)
	Day 1	Day 2		
Breathing exercises	Hands stretch breathing	Hands stretch breathing	—^b^	10 min
	Hands in and out breathing	Hands in and out breathing	9	
	Yogic breathing	Yogic breathing	9	
	Ankle stretch breathing	Ankle stretch breathing	9	
	*Kapalabhati kriya*	*Kapalabhati kriya*	9	
	Yogic breathing	Yogic breathing	3	
Asanas: standing	*Surya namaskar*	*Surya namaskar*	4	6 min
	*Ardha kati chakrasana*	*Trikonasana*	1	10 min
	*Padahasthasana*	*Parshvakonasana*	1	
	*Ardhachakrasana*	*Vrikshasana*	1	
	*Trikonasana*	*Veerabhadrasana*	1	
Asanas: supine	*Shavasana*—quick relaxation technique	*Shavasana*—quick relaxation technique	1	5 min
	*Uttanapadasana*	*Pawanamuktasana*	1	5 min
	*Sarvangasana*	*Suptapadangushtasana*		
	*Matsyasana*	*Viparaitakarni*		
Asanas: prone	*Bhujangasana*	*Dhanurasana*	1	4 min
	*Shalabhasana*	*Makarasana*		
Asanas: sitting	*Shashankasana*	*Ardhamatsyendrasana*	1	4 min
	*Ustrasana*	*Gomukhasana*		
Pranayama	*Nadishuddi*	*Nadishuddi*	9	6 min
	*Bhramari*	*Bhramari*	9	
Meditation or relaxation	*Om* meditation or guided meditation	*Om* meditation or guided meditation	1	10 min
	*Shavasana*	*Shavasana*	1	

a Total duration: 60 min.

b Not applicable.

The participants in the control group (CG) followed the existing AS for high altitude. The research team, comprising a medical expert, an Ayurveda practitioner, and a yoga instructor, administered the Ayush interventions at the study sites throughout the study. The participants were monitored at frequent intervals at both study sites.

Ayush CCT was provided by the Indian Medicines Pharmaceutical Corporation Limited, India, and Ayush poshak yoga by MARC Laboratories Ltd, India.

### Discontinuation of the Study Interventions

If any participant developed adverse effects, such as gastrointestinal symptoms, allergic reactions, or changes in biochemical assessment parameters, the administration of the Ayur-nutri kit was temporarily halted, and the participant was closely monitored. If symptoms recurred after reintroducing the nutritional supplements, the participant was withdrawn from the study after assessing the causality of adverse event (AE) or adverse drug reaction. All such events were recorded and reported in the AE or adverse drug reaction reporting format.

### Compliance With the Study Interventions

All participants in the AG group were provided with an information leaflet containing instructions for the use (dose, frequency, and time of administration) and storage of the Ayur-nutri kit. The participants were also issued a compliance form during the baseline visit and subsequent follow-up visits to self-report their consistent or irregular use of Ayush interventions in the Ayur-nutri kit and to record any missed doses, along with remarks for missing doses, which enabled the assessment of adherence to the dosing pattern as per the study protocol. During each follow-up visit, the participants were asked to return the used, unused, or partially used sachets of the Ayur-nutri kit to the investigators to assess adherence and cross-check with the participant’s self-reported compliance form. The participants were advised to maintain an open communication channel with the research team to address any issues they faced, including missed doses, adverse effects, and general health-related queries.

Adherence to the 60-minute daily yoga protocol was systematically monitored through instructor-maintained attendance logs at each site and also from the participant’s compliance diary. Daily session attendance was recorded for every participant throughout the intervention period.

The participants who did not adhere to the study protocol, or did not have 80% or more compliance, or those who developed any study-related AEs due to which the participant preferred to withdraw from the study, or those who withdrew their voluntary consent for participation in the study, were withdrawn from the study. If participants consented to data collection during the scheduled follow-ups or at the end of the 120 days, data were collected and recorded in the case report form (CRF). The data from completed assessments, available until the withdrawal, were used for analysis.

### Concomitant Care

The investigators monitored the participants for any concomitant treatment they received during the study period. In the event of any AEs, the use of rescue medication was permitted under the investigator’s discretion. All instances of concomitant care were carefully documented in the CRF. However, any study participant prescribed a conventional medication for a longer duration (more than 6 consecutive days) during the study period must discontinue use of the Ayush intervention during that period. This threshold distinguishes brief, incidental, or rescue medication use from sustained therapy that could potentially interact with or confound the effects of the study intervention. All such instances were documented in the CRF. If any participant developed a serious AE or a treatment-emergent AE during the study period, the participant was withdrawn from the study and given appropriate incidental care at the hospital. The government authorities and the Ethics Committee were notified within 2 working days, along with an appropriate justification.

### Outcome Measures

The primary outcome was the incidence of AMS, assessed using the 2018 Lake Louise Scoring System (LLSS). AMS is defined as an LLSS score of 3 or more points from the 4 rated symptoms (headache, nausea or vomiting, fatigue, and dizziness or light-headedness), including at least 1 point from headache [9]. The grading of LLSS score is mild AMS 3‐5 points, moderate AMS 6‐9 points, and severe AMS 10‐12 points [9]. The participants were assessed daily for the first 7 days at the stage I high-altitude areas using the LLSS score to record the incidence and evaluate the severity of AMS. The primary outcome will be reported as the proportion of participants who display symptoms of AMS and the proportion of participants with a score of more than 6 as per the LLSS. The participants enrolled for stage II were again assessed daily during their first 4 days at that altitude for the incidence and severity of AMS.

The secondary outcome measures included the proportion of participants who developed thromboembolic events (deep-vein thrombosis, pulmonary embolism, cerebral venous thrombosis, myocardial infarction, etc) during the study, change in the levels of markers of coagulation and hemostasis activation (total red blood cell count, hemoglobin, packed cell volume, D-dimer, lactate dehydrogenase, creatine kinase, creatine kinase-myocardial band, fibrinogen, activated partial thromboplastin time, prothrombin time), changes in the levels of proinflammatory markers (high sensitivity C-reactive protein, interleukin-6, tumor necrosis factor-α); self-reported changes in negative emotional states of depression, anxiety, and stress assessed through Depression Anxiety Stress Scale-21 item (DASS-21); change in sleep quality assessed through the Pittsburgh Sleep Quality Index (PSQI), and quality of life assessed through the 12-item short-form (SF-12) questionnaire. The secondary outcomes were evaluated on day 60 and day 120 from baseline. However, the participants enrolled for the stage II high-altitude area were also assessed on day 10 (day 4 of the AS at that altitude) and on the day of descent, in case of early descent before day 120.

Resting heart rate, systolic and diastolic blood pressure, respiratory rate, and oxygen saturation (SpO2) were also documented daily until day 7, alongside LLSS at stage I high altitude, and until day 10 in participants at stage II high altitude. These measurements were then taken on scheduled follow-up visits.

### Safety Assessment

The safety of the Ayush interventions was determined by recording the incidence of AEs, if any, during scheduled follow-up visits in a structured format. All AEs during the study were recorded and monitored as per ICH (International Council for Harmonisation of Technical Requirements for Pharmaceuticals for Human Use) Good Clinical Practice guidelines. Safety was also evaluated by assessing liver function tests, kidney function tests, and lipid profiles on days 60 and 120 from baseline.

### Sample Size

Based on the findings from a previous study on similar conditions, the anticipated incidence of AMS within the CG was 25%, while the assumed incidence in the AG was 18% [7]. To achieve a statistical power of 90% and a CI of 95%, an initial sample size of 722 per group was calculated. Accounting for an expected attrition rate of 15%, the final sample size for each group was determined to be 830 participants (N=1660 in total). The sample size of 1660 participants was stratified into stage I high altitude and stage II high altitude. A total of 830 participants were included from stage II high altitude, while the remaining 830 were from stage I high altitude. The participants studied at each of the 2 altitude stages were randomly allocated to the AG and CG.

### Recruitment of Study Participants

During the study, the database of healthy personnel in high-altitude areas of the western Himalayan region was used to identify and recruit potential subjects. The investigators at the study site screened subjects based on the defined inclusion and exclusion criteria. The eligible participants were allocated to either of the 2 groups based on a computer-generated randomization schedule. The screening process was continued until the target sample size for the study was achieved.

### Randomization

Block randomization with unequal block sizes randomly assigned participants to either the AG or CG with a 1:1 allocation to minimize predictability. Block randomization was implemented separately for stage I and stage II participants to ensure balanced allocation within each altitude-exposure setting. The SPSS version 28.0 (IBM) was used to generate the random number sequences. An independent statistician not involved in the participants’ enrollment and assessment generated the randomization sequence.

### Allocation Concealment

Sequentially numbered, opaque, sealed envelopes were used to ensure allocation concealment. The participant’s enrollment number was printed at the top of the envelope, and a slip indicating the participant’s allocated group was kept inside. After completing all baseline assessments, the research staff provided the sealed envelope to the eligible participant. The participant opened the envelope and was then assigned to the group as indicated on the slip inside. The opened envelope and the printed slip were attached to the participant’s CRF for record and trial monitoring.

Participants assigned to the 2 study groups were housed in separate barracks throughout the study to prevent any protocol violations and minimize the risk of contamination bias. Further, yoga sessions and supplement distribution were conducted at designated areas accessible only to the intervention group, with attendance monitored by site instructors. The CG did not receive access to these sessions or materials, reducing the likelihood of unintentional participation. Study staff members were instructed to avoid sharing intervention-related information with control-group participants.

### Blinding

The study is open-label; however, to minimize any potential bias, the research staff assigned to assess the study outcomes will be blinded to the allocation of participants to the study groups. In addition, laboratory technicians analyzing blood samples were blinded through the use of coded specimens. The intervention team (Ayurveda practitioner and yoga instructor) had no role in outcome assessments, data entry, or laboratory procedures. The assessment team functioned independently and interacted with participants only at scheduled assessment time points. They were instructed not to inquire about participants’ allocation or intervention experiences. Each participant was assigned a unique study ID; all CRFs, questionnaires, and lab requisitions contained only this ID and not the allocation label. Further, statistical analysis will be conducted by an independent data analysis team not involved in intervention delivery or participant contact.

### Data Collection

The baseline demographics, clinical, and physical examination aspects were collected by qualified Ayush study personnel and reported in a CRF designed for this purpose. Subjective and objective outcome assessments were conducted according to the protocol (Table 3).

**Table 3.** Study schedule.

	Screening	Baseline	Day 1 to 7	Day 10 (for participants at stage II HA^a^)	Day 30	Day 60	In case of early descent	Day 120
Written informed consent	✓							
Eligibility evaluation	✓							
Demographics and medical history		✓						
Clinical assessment	✓	✓	✓	✓	✓	✓	✓	✓
Vital parameters (resting heart rate, systolic and diastolic blood pressure, respiratory rate, SpO_2_^b^)	✓	✓	✓	✓	✓	✓	✓	✓
CBC^c^	✓			✓		✓	✓	✓
LFT^d^, KFT^e^	✓					✓	✓	✓
HbA_1c_^f^ and ECG^g^	✓							
Lipid profile		✓				✓	✓	✓
LDH^h^, CK^i^, CK-MB^j^, fibrinogen, aPTT^k^, prothrombin time, hs-CRP^l^, IL-6^m^, TNF-α^n^		✓		✓		✓	✓	✓
Lake Louise Scoring System for AMS^o^ assessment		✓	✓	✓				
Assessment for thromboembolic events						✓	✓	✓
DASS-21^p^, PSQI^q^, SF-12^r^ questionnaire		✓		✓		✓	✓	✓
Rescue medication			✓	✓	✓	✓	✓	✓
Assessment of adverse events			✓	✓	✓	✓	✓	✓
Assessment of drug compliance			✓	✓	✓	✓	✓	✓

a HA: high altitude.

b SpO_2_: peripheral capillary oxygen saturation.

c CBC: complete blood count.

d LFT: liver function test.

e KFT: kidney function test.

f HbA_1c_: glycated hemoglobin.

g ECG: electrocardiogram.

h LDH: Lactate Dehydrogenase.

i CK: creatine kinase.

j MB: myocardial band.

k aPTT: Activated Partial Thromboplastin Time.

l hs-CRP: high-sensitivity C-reactive protein.

m IL-6: interleukin-6.

n TNF-α: tumor necrosis factor-alpha.

o AMS: acute mountain sickness.

p DASS-21: Depression Anxiety Stress Scale-21 items.

q PSQI: Pittsburgh Sleep Quality Index.

r SF-12: 12-item short-form.

The incidence of AMS and its severity were assessed using the LLSS AMS score, according to which AMS is diagnosed by having a headache score of at least 1 point and a total score of at least 3 points [9]. This was assessed daily for the first day in participants at stage I high altitude and for an additional 4 days in participants who progressed to stage II high altitude. The self-reported emotional states of depression, anxiety, and stress were reported by adding scores of the relevant items in the 3 subscales of the DASS-21 [28]. The quality of sleep and overall quality of life were assessed through validated scales, the PSQI and the SF-12 questionnaire, respectively [29-34]. The secondary outcomes, including DASS-21, PSQI, and SF-12, were assessed at baseline and at follow-ups on days 60 and 120.

The serum sample for the objective assessment parameters, such as coagulation and hemostasis activation markers, proinflammatory markers, LFT and RFT, etc, was collected at baseline, day 60, and day 120 and transported to an NABL-accredited laboratory, and the data received from the lab were entered into the CRFs and electronic CRFs. The research team underwent training on the study protocol, standard operating procedures for the conduct of the study, storage, and dispensing of interventions, handling of biological samples, data collection, and recording to ensure compliance with GCP principles while ensuring participant safety, data accuracy, and reliability.

### Data Management

Data management in this clinical study adhered to stringent guidelines to ensure the accuracy, reliability, and integrity of the collected information. Upon the participants’ assessments, the research team promptly entered the data into CRFs and electronic CRFs. Source documents and CRFs were securely stored in restricted-access areas, limited solely to the study team. Electronic CRFs were password-protected and stored in secure, access-restricted computer systems.

Data entered by the study personnel was subjected to meticulous cross-verification by the principal investigators at the study site, ensuring the reliability of the data. Rigorous quality measures were implemented, such as regular audits, to identify and address any discrepancies in the data. The data management practices adhered to regulatory guidelines and ethical principles, prioritizing the protection of participants’ rights.

### Statistical Methods

The categorical data will be presented as numbers (percentages) and compared between groups using the *χ*^2^ test, while within-group comparisons will be performed by using the McNemar or Cochran Q-test. Continuous data following a normal distribution will be reported as mean (SD), and between-group comparisons will be performed using an independent sample *t*-test. Within-group comparisons for normal data will be performed using a paired sample *t*-test/repeated measures ANOVA. Nonnormal data will be reported as median (first quartile, third quartile), and between-group comparisons will be conducted using the Mann-Whitney test. Within-group comparison will be done using the Wilcoxon signed-rank test or Friedman test. Baseline variables, such as age, prior altitude exposure, and hydration-related indicators, will be examined descriptively and adjusted in multivariable models where relevant. Regression analysis will be performed to study the effect of confounding variables on the incidence of AMS. The data of participants having early descent will be analyzed with censored data models. A *P* value less than .05 will be considered significant. The SPSS version 28.0 is being used for statistical analysis.

The modified intention-to-treat analysis approach will be applied to handle missing data. The missing data for all participants for whom data are available for at least one visit post-baseline will be imputed. The last observation carried forward method will be used for imputing the missing values.

### Monitoring

The Data and Safety Monitoring Board monitored the study for quality and regulatory compliance. The Data and Safety Monitoring Board reviewed the study’s progress every 3 months until the end of the study period.

### Trial Audit

The on-site monitoring visit by an independent committee constituted by the sponsor was planned to ensure that the study procedures and data collection processes adhered to existing regulatory standards and to verify the accuracy, completeness, legibility, and timeliness of the reported study data.

### Ethical Considerations

The Institutional Ethics Committee has approved the study protocol to ensure compliance with ethical standards and safeguard the rights and well-being of the participants. The study has been registered prospectively at the Clinical Trial Registry of India (CTRI/2023/03/051028). The study was conducted in accordance with the principles of the Declaration of Helsinki, the ICMR’s National Ethical Guidelines for Biomedical and Health Research on Human Participants (2017) [35], and the ICH Good Clinical Practices guidelines [36]. No substantial amendments in the study protocol were made that may affect patient safety or study integrity. Before undergoing any study-related procedure, potential participants received a participant information sheet in their native language or Hindi. The participant information sheet comprehensively outlined the various aspects of the study, equipping participants with the necessary information to make an informed decision about participating in the study. Written consent was obtained through the consent form, which was signed by both the participant and the study personnel delegated to the task. No financial compensation will be provided to participants for their involvement in the study. To ensure participant safety, clinical trial insurance has been secured to cover any unexpected adverse events or study-related injuries.

The results and findings of the study will be disseminated in accordance with best practices in scientific publishing, ensuring that the knowledge generated benefits the scientific community and the public at large through publication in peer-reviewed, indexed medical journals, and presentations at national and international conferences.

### Confidentiality

All the relevant study data were stored securely at the study site with password-protected access systems in areas with limited access. To maintain participant confidentiality, a coded enrollment identification number was used to identify all laboratory specimens, reports, data collection, and relevant forms. All records containing names or other personal identifiers, such as informed consent forms, were stored separately from the study records and identified by a code number in a restricted-access area.

### Ancillary and Posttrial Care

No ancillary studies were proposed for the present clinical study. If required, participants were provided with routine medical care after completing the study period.

## Results

The study was funded in March 2023. The data collection was completed in December 2023. A total of 1660 participants were enrolled in the study. The analysis of the study data is in progress. The study outcomes are expected to be published by December 2026.

## Discussion

### Novel Aspects of the Study

High-altitude regions pose unique physiological and psychological challenges. The present publication outlines a groundbreaking RCT designed to assess the impact of holistic Ayush interventions, consisting of Ayurvedic nutritional supplements (Ayur-nutri kit) and Yoga, in conjunction with the existing AS, on reducing the incidence of AMS, while also addressing hemostatic and vascular function, psychological well-being, and overall quality of life among personnel at high altitude.

A conventional AS is routinely followed to prevent HAI. It is primarily based on a strategy that combines the conventional staged and graded ascents practiced by mountaineers [27]. The 3-stage AS extends up to 14 days to reach stage III high-altitude regions (more than 15,000 feet) [37]. Stage I acclimatization lasts for 6 days for stage I high altitude (9000‐12000 feet), whereas an additional 4 days each are required to achieve stage II (12,000‐15,000 feet) and stage III (more than 15,000 feet) acclimatization [27,37]. During reinduction to high-altitude regions, after 10 to 30 days of absence from high altitude, 4 days at each stage need to be spent, while in the case of more than 30 days, a full AS as a fresh inductee needs to be complied [37]. However, effective interventions for preventing HAI with a better safety profile must be explored to facilitate better acclimatization, shorter ascent times to reach high-altitude regions, and optimal use of limited resources. It could also have logistical implications, such as the early availability of healthy personnel for high-altitude regions.

The preliminary evidence suggests that the proposed Ayush interventions, viz Ayurvedic nutritional supplements and yoga protocols are safe and can potentially alleviate the pathophysiology of HAI, aid in better acclimatization, and reduce the incidence and severity of AMS [22-25]. Furthermore, the herbal ingredients of the Ayur-nutri kit possess cardioprotective, antidyslipidemic, antihypertensive, anti-inflammatory, antiatherogenic, antiplatelet aggregation, antithrombotic, and anticoagulant properties [17-21]. Therefore, these supplements may also have potential benefits in maintaining healthy circulation, thereby possibly reducing thromboembolic events at high altitude by showing favorable effects on markers of coagulation and hemostasis activation, as well as proinflammatory markers. The integration of nuts and seeds in the Ayur-nutri kit provides nutrient-dense nourishment that ensures an adequate supply of essential vitamins, minerals, and healthy fats, while serving as an excellent energy source, providing sustained fuel for physical activities and supporting cardiovascular health. The 60-minute yoga protocol, tailored for high altitude, can offer a wide range of benefits to enhance resilience and promote physical and psychological well-being. Yoga cultivates mental and emotional well-being, which is vital for personnel coping with challenging and unpredictable high-altitude conditions. Meditation and relaxation techniques can improve sleep, which is often disrupted at high altitudes. Better sleep quality supports cognitive function, mood stability, and overall well-being. A well-structured Ayush-AMS protocol that addresses the unique needs and challenges of high altitude can promote holistic well-being.

The present RCT represents an innovative approach by incorporating a holistic strategy in mitigating the challenges posed by AMS and the various physiological and psychological stressors associated with high altitude. The comprehensive Ayush-AMS regimen has the potential to inform future protocols in HAI and offer valuable contributions to the broader field of high-altitude medicine.

### Strengths and Limitations

The present RCT protocol has particular key strengths. First, the use of nutritional supplements enriched with Ayurveda herbs, which are easy to use, palatable, and safe, with the potential to address AMS and related conditions. Second, the study has a large sample size, validated subjective assessment tools, and biomarkers to address various circulation and hemostasis-related parameters. This protocol also has a few limitations, including the open-label design of the study. However, to remove any potential bias, the research staff assigned to assess the study outcomes will be unaware of the allocation of participants to the study groups. Furthermore, there is an unequal attention between groups (due to oral interventions and supervised yoga routines in the AG) that may influence participants’ perceptions. Additionally, the altitude-exposure stage may influence the onset patterns of symptoms. Also, as the study is exploratory in nature for the secondary outcomes, it lacks power for multiple hypothesis testing across biomarkers, and no hierarchical testing plan or multiplicity adjustment was prespecified.

### Expected Outcomes

The outcomes of the present RCT are expected to facilitate the optimal use of limited resources through better acclimatization, reduced ascent time, and the rapid induction of healthy personnel at high-altitude regions during exigency situations.

## Abbreviations

AE: adverse event
AG: Ayush group
AMS: acute mountain sickness
AS: acclimatization schedule
CCRAS: Central Council for Research in Ayurvedic Sciences
CCT: cardiac care tea
CG: control group
CRF: case report form
HAI: high-altitude illness
HAPE: high-altitude pulmonary edema
ICH: International Council for Harmonisation of Technical Requirements for Pharmaceuticals for Human Use
LLSS: Lake Louise Scoring System
PSQI: Pittsburgh Sleep Quality Index
RCT: randomized controlled trial
SF-12: 12-item short-form
SPIRIT: Standard Protocol Items: Recommendations for Interventional Trials
